# Therapeutic Notes

**Published:** 1898-06-04

**Authors:** 


### THERAPEUTIC NOTES.

### Diarrhœa: A Rough Rule.

The following rule is given by Dr. Seymour Taylor as being a very useful one in prescribing for obstinate diarrhœa: So long as the tongue is not abnormally red or beefy—that is, so long as there is a fair amount of fur upon it—alkalies and vegetable astringents are the most effective; but if after a time the diarrhœa continues and the tongue becomes glazed and raw, I find it best to give mineral acid and opium with it.

